# Overproduction of *Bacillus amyloliquefaciens *extracellular glutamyl-endopeptidase as a result of ectopic multi-copy insertion of an efficiently-expressed *mpr *gene into the *Bacillus subtilis *chromosome

**DOI:** 10.1186/1475-2859-10-64

**Published:** 2011-08-05

**Authors:** Yurgis AV Yomantas, Elena G Abalakina, Lyubov I Golubeva, Lyubov Y Gorbacheva, Sergey V Mashko

**Affiliations:** 1 Ajinomoto-Genetika Research Institute, 117545, Moscow, Russian Federation

## Abstract

**Background:**

Plasmid-less, engineered *Bacillus *strains have several advantages over plasmid-carrier variants. Specifically, their stability and potential ecological safety make them of use in industrial applications. As a rule, however, it is necessary to incorporate many copies of a key gene into a chromosome to achieve strain performance that is comparable to that of cells carrying multiple copies of a recombinant plasmid.

**Results:**

A plasmid-less *B. subtilis *JE852-based strain secreting glutamyl-specific protease (GSP-the protein product of the *mpr *gene from *B. amyloliquefaciens*) was constructed that exhibits decreased levels of other extracellular proteases. Ten copies of an *mpr^B.amy ^*cassette in which the GSP gene was placed between the promoter of the *B. amyloliquefaciens rplU-rpmA *genes and the Rho-independent transcription terminator were ectopically inserted into designated (3 copies) and random (7 copies) points in the recipient chromosome. The resulting strain produced approximately 0.5 g/L of secreted GSP after bacterial cultivation in flasks with starch-containing media, and its performance was comparable to an analogous strain in which the *mpr^B.amy ^*cassette was carried on a multi-copy plasmid.

**Conclusion:**

A novel strategy for ectopically integrating a cassette into multiple random locations in the *B. subtilis *chromosome was developed. This new method is based on the construction of DNA fragments in which the desired gene, marked by antibiotic resistance, is sandwiched between "front" and "back" portions of random chromosomal DNA restriction fragments. These fragments were subsequently inserted into the targeted sites of the chromosome using double-cross recombination. The construction of a marker-free strain was achieved by gene conversion between the integrated marked gene and a marker-less variant carried by plasmid DNA, which was later removed from the cells.

## Background

Gram-positive bacteria are widely used for biotechnology applications, including vaccine delivery [1-3] and *in situ *production of anti-infective protectants [4] and microbicides [5]. These microorganisms serve as large-scale producers of nucleotides, vitamins, ribose, poly-γ-glutamic acids [6], absorbents [7], and insecticides [8]. *Bacillus *species are considered prospective cell-based factories for pharmaceutical proteins [9]. Currently, about 60% of commercially-available industrial enzymes are produced by selected and/or genetically-engineered *Bacillus *strains, most of which produce homologous proteins that are naturally secreted into the growth medium [6,9-15].

*Bacillus subtilis *produces numerous extracellular proteolytic enzymes. The alkaline serine protease subtilisin and the neutral protease (gene products of *aprE *and *nprE*, respectively) often constitute more than 90% of the total extracellular protease activity [9,16]. The contribution of glutamic acid-specific protease (GSP) does not normally exceed 2% [17]. *B. subtilis *GSP, encoded by the *mpr *gene, is synthesized as an inactive pre-pro-peptide. This precursor is subsequently processed by the Sip and Bpr proteases, and mature extracellular GSP have a length of 220 amino acids [17]. Though they were initially a subject of basic science investigation [18-20], some GSPs (from *B. licheniformis *in particular [21]) are now being utilized in commercial applications such as food production [22,23].

A traditional approach to the genetic engineering of *Bacillus *strains involves the introduction of multi-copy-number recombinant plasmids [10]. However, the construction of plasmid-less strains has recently become more relevant and practical. The preference for plasmid-less *Bacillus *strains is due to the genetic instability of many recombinant plasmids [24,25] and to official restrictions that concern the use of plasmid-carrying strains in large-scale industry in the First World [26]. Most often, the construction of plasmid-less *Bacillus *strains is performed by homologous recombination-mediated integration of the desired genes into the bacterial chromosome [10]. In some instances, specialized site-specific recombination [27] and transposition [28,29] are used for the same integrative purposes.

Recombination-mediated DNA incorporation can be implemented through either Campbell-type single-crossover integration of plasmids based on specialized vectors carrying DNA sequences homologous to the *Bacillus *chromosome or through the use of ectopic insertion, i.e. double-cross recombination between the target in the chromosome and the homologous flanking sequences sandwiching the fragment of interest [10,30,31]. Both methods can be used for single-copy and multi-copy integrations [32-34]. Single-copy, plasmid-mediated integrants with inserted sequences bracketed by duplicated homologous regions are not stable under non-selective conditions due to the possible recombination-mediated elimination of the inserted plasmid [35]. Ectopic insertion(s) of a desired gene usually leads to significantly more stable recombinant strains. However, only a narrow set of well-characterized loci within the *B. subtilis *chromosome is normally used as targets for such insertions [10,36,37].

In this study, a recombinant, plasmid-less *B. subtilis *strain was developed that can efficiently produce and secrete GSP from *B. amyloliquefaciens*. Initially, three copies of the *mpr *gene were ectopically inserted into known *B. subtilis *genes encoding extracellular proteases. A novel, random integration methodology was then implemented to construct a stable strain with 10 *mpr *copies within the chromosome. Performance of the new strain was comparable to the strain carrying the *mpr *gene on a multi-copy plasmid, as exhibited by accumulation of the recombinant GSP in the media.

## Results

### Cloning and expression of *B. amyloliquefaciens mpr *on a *B. subtilis *plasmid

The nucleotide sequence of the *mpr *gene from *B. amyloliquefaciens *A-50 was not known. Primers for the amplification of *mpr *by PCR, mpr-F/R (the structures of the primers used in this study were presented in Additional file 1, **Table S1**), were therefore designed based on the available *B. amyloliquefaciens *ZB42 genome sequence (GenBank/EMBL accession number NC_009725) [38]. DNA amplicons of 972 base pairs (bp) in length were obtained and sequenced (GenBank accession number GU992366). The corresponding DNA sequence closely coincided with the *mpr-*containing sequence from *B. amyloliquefaciens *FZB42 (91% of identity) and covered the 909-bp open reading frame. An extended AG-rich block, including a *B. subtilis *Shine-Dalgarno sequence, AAGGAGG [39], was found upstream of the ATG codon of this ORF. The protein-coding ORF possessed 68% identity to well-characterized pre-pro-GSP from *B. subtilis *[17,18,20].

**Figure 1:**
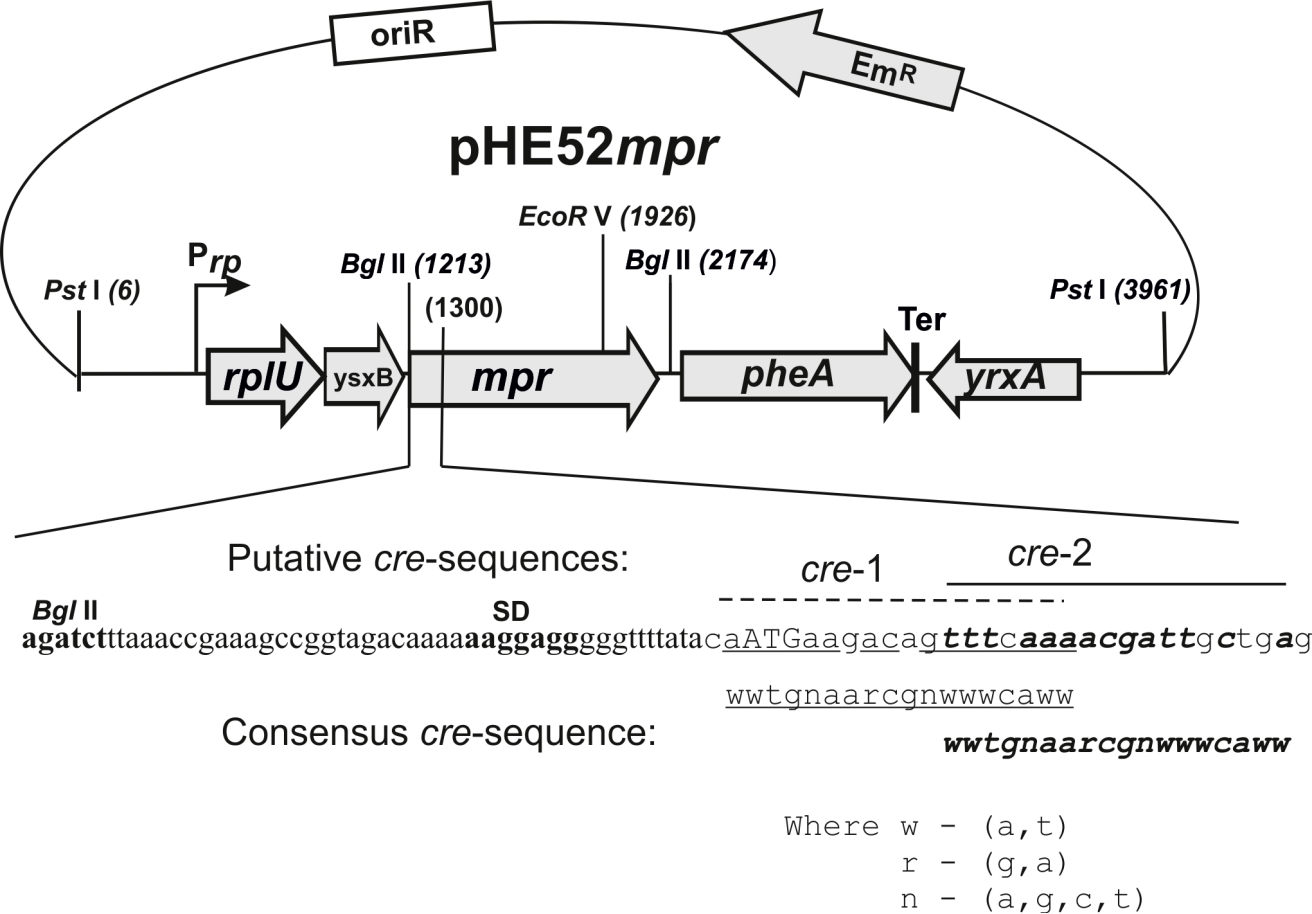
**Structure of the pHE52*mpr *plasmid carrying the *mpr^B.amy ^*cassette**. P*_rp _*- promoter of the *rplU-rpmA *genes from *B. amyloliquefaciens *A-50; Ter - Rho-independent transcription terminator of the *pheA *gene. The *Bgl*II-site-ended (boldface) followed by 5'-portion of *B. amyloliquefaciens *A-50 *mpr *gene is shown. In this part, SD-sequence and ATG-codon are marked as boldface and capital letters, respectively. Two putative *cre *elements, which are homologous to the known [45-47] consensus sequence, are also indicated.

The *mpr*-carrier amplicon, flanked by artificial *Bgl*II sites (P1-bmp5 and P2-bmp2 were used as the primers), was cloned into the *Bgl*II site of the pHEA323 plasmid [40]. This placed it under the transcriptional control of the promoter (P*_rp_*) of the *rplU-rpmA *genes from *B. amyloliquefaciens *A-50, which encode the L21 and L27 ribosomal proteins. In the resulting pHE52*mpr *recombinant plasmid, the cloned *mpr *gene became the central part of an artificial operon that was terminated by the Rho-independent transcription terminator (Ter) from the *pheA *gene of *B. amyloliquefaciens *A-50 (Figure 1). As was shown previously [40] and confirmed in the present study, the presence of Ter for the termination of efficient P*_rp_*-mediated transcription is conducive to the stable inheritance of pHEA323 and its derivatives (i.e., *mpr^B.amy ^*cassettes (P*_rp_*➔*mpr*-Ter) in the pHE52*mpr *plasmid and/or integrated into the bacterial chromosome).

*Mpr *gene expression studies were performed with the *B. subtilis *strain JE852 serving as a recipient. This strain was a double mutant for genes encoding two major extracellular proteases (*nprE512, aprE851*), which simplified the assessment of recombinant GSP activity.

Initially, a plasmid-carrying, recombinant GSP-producing strain was constructed via the transformation of *B. subtilis *JE852 with pHE52*mpr*. The level of GSP accumulation was analyzed by the semi-quantitative skim milk method on media containing different carbon sources and by sodium dodecyl sulfate-polyacrylamide gel electrophoresis (SDS-PAGE) analysis of extracellular proteins. It was shown in these experiments that the expression of the *mpr^B.amy ^*cassette was under carbon catabolite control (CCC) in *B. subtilis*. Indeed, when glucose or maltose were added to the media, *B. subtilis *JE852/pHE52*mpr *grew well but did not form clear, hydrolytic zones around colonies on milk agar. On the other hand, during growth on medium containing soluble starch as the sole carbon source, abundant amounts of GSP accumulated and were easily distinguished from the other extracellular proteins by SDS-PAGE. The main mechanism of CCC in *Bacillus *has been well studied [41-44]. CCC is implemented through the binding of the CcpA-mediated regulatory protein complex to special DNA sites known as catabolite responsive elements (*cre*). This binding causes carbon catabolite repression (CCR) or activation (CCA), depending on the position of the *cre*. For example, when the regulatory complex binds to *cre *that is located downstream of the transcription initiation point, it evokes a transcription roadblock that leads to CCR of the corresponding genes [44]. Two putative catabolite responsive elements that were homologous to consensus *cre *sequences [44-46] were found in the N-terminal coding part of the *B. amyloliquefaciens *A-50 *mpr *gene by sequence analysis (Figure 1). It is possible that the CCR of *mpr *gene expression that we observed was caused by termination of transcription at these *cre *sites when they were bound to the CcpA-mediated regulatory complex. Moreover, the data suggest that a complicated regulatory network governs *Bacillus *extracellular proteolytic activity with CCR and that there may be changes in the control of enzyme biosynthesis, secretion, and/or maturation at different stages of bacterial growth [41,42,47].

Defining the mechanism of CCR modulation of GSP extracellular accumulation was outside the scope of the present paper. We showed that GSP production was significantly increased during fermentation of the *B. subtilis *JE852/pHE52*mpr *strain on TYS6C media, in which starch was the main carbon source. In this media, an enhanced biomass (growing up to an OD_600 _of around 40-50) and high level of extracellular GSP accumulation (up to approximately 0.5 g/L, as semi-quantitatively determined by SDS-PAGE, see **Materials and methods**) were detected. These results were obtained for the strain carrying multi-copy-number recombinant plasmids, suggesting that the integration of multiple copies of the *mpr^B.amy^*-cassettes into the bacterial chromosome is indispensable for achieving comparably high GSP production levels in a plasmid-less *Bacillus *strain.

### Ectopic insertion of *mpr^B.amy ^*cassettes into genes encoding known extracellular proteases

**Figure 2:**
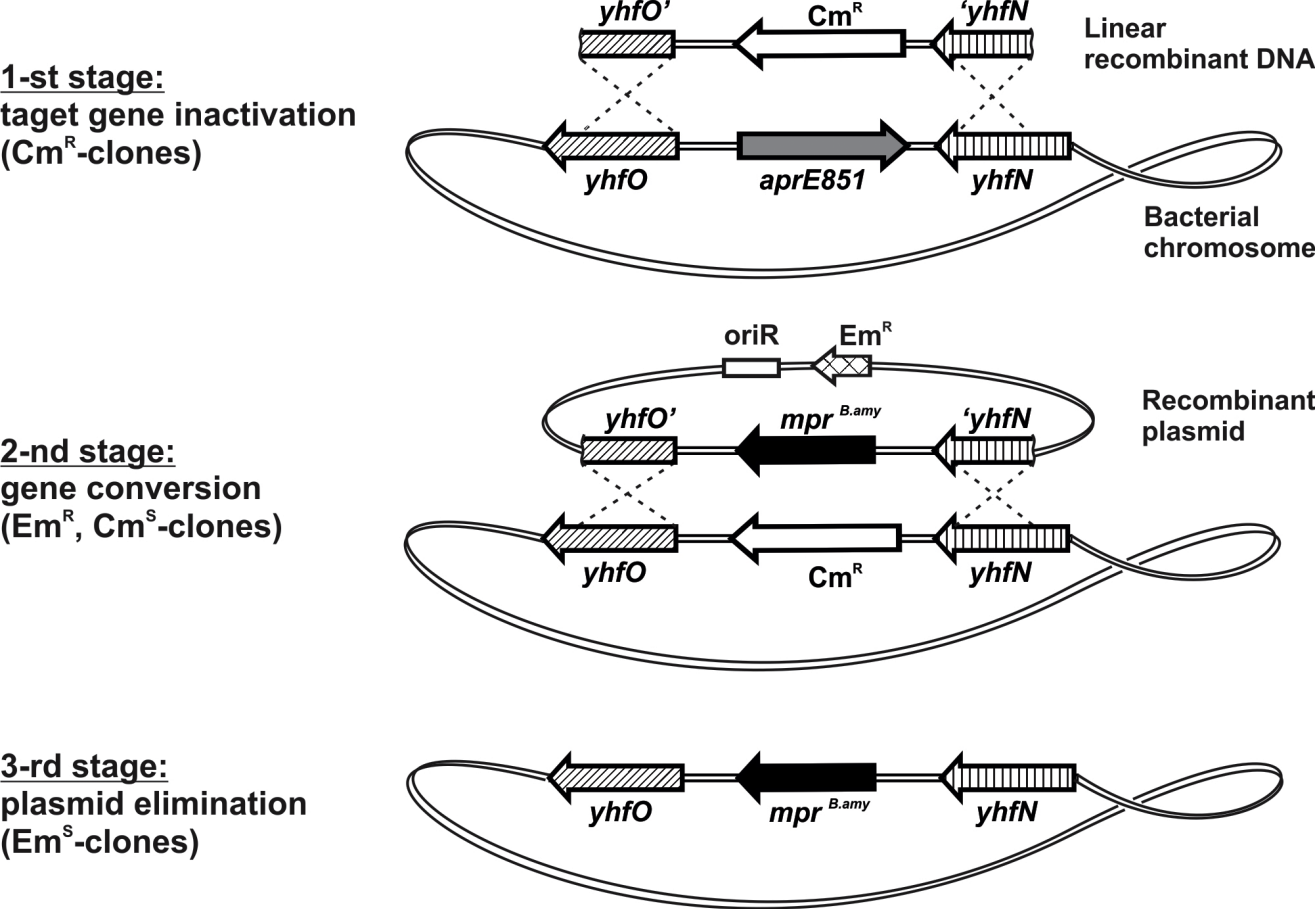
**Scheme of the targeted *mpr^B.amy^*-cassette integration into the JE852 chromosome (*aprE851 *is shown as an example of the target gene)**.

Ectopic insertion of several *mpr^B.amy ^*cassettes was performed to simultaneously inactivate known extracellular protease genes of *B. subtilis*: *aprE, epr *and *nprB*. The overall scheme of *mpr^B.amy ^*cassette insertion had three stages (see Figure 2 where the *mpr^B.amy ^*cassette insertion into the *aprE851 *allele of *B. subtilis *JE852 strain is shown as an example). First, a linear DNA fragment consisting of an antibiotic resistance (AntR) marker flanked with homologous arms was integrated into the corresponding chromosomal region via double-crossover recombination. Then, the AntR marker was exchanged for the *mpr^B.amy ^*cassette by gene conversion (for a review, see [48,49]) between the chromosome and the autonomously replicating *mpr^B.amy^*-carrying plasmid, and this was followed by plasmid curing and construction of the plasmid-less, targeted integrant.

The *aprE851 *gene in *B. subtilis *JE852 was chosen as the first target gene for *mpr^B.amy ^*cassette insertion, primarily to prevent reversion of the mutant allele to the wild-type phenotype during the proposed long-term construction of a GSP-producing, plasmid-less strain. The Cm^R ^gene from pC194 [50] was used as the AntR marker for selective integration at the first stage. The linear fragment, used for *aprE851 *gene disruption, was constructed *in vitro *by overlapping PCR technique (see **Materials and methods **and Additional file 2, **Figure S1 **for details). The targeted integration of the Cm^R ^marker was followed by gene conversion using the Em^R^-marked recombinant plasmid pCBT(*yhfO*-*mpr^B.amy^*-*yhfN*) and subsequent selection of the obtained Em^R ^Cm^S ^clones, which were generated at a frequency of around 2%. Finally, the plasmid-less (Em^S^) variants were selected after bacterial cultivation in liquid erythromycin-free medium. All integration stages were assessed by PCR, and the chromosome structure of the *B. subtilis *JE852*aprE851*::*mpr^B.amy ^*strain was analyzed by PCR and using Southern hybridization.

The same method, with modifications based on the nucleotide sequences of the target genes, was used for step-by-step ectopic insertion of the *mpr^B.amy ^*cassette into the *epr *and *nprB *genes, encoding two minor extracellular proteases of *B. subtilis *(see **Materials and methods **and Additional file 1, **Table S1 **for details). This process resulted in the desired *B. subtilis *strain, a JE852-based plasmid-less, marker-less strain, JE852(*aprE851, epr, nprB*)::*mpr^B.amy^*, with three integrated *mpr^B.amy ^*cassettes.

**Figure 3:**
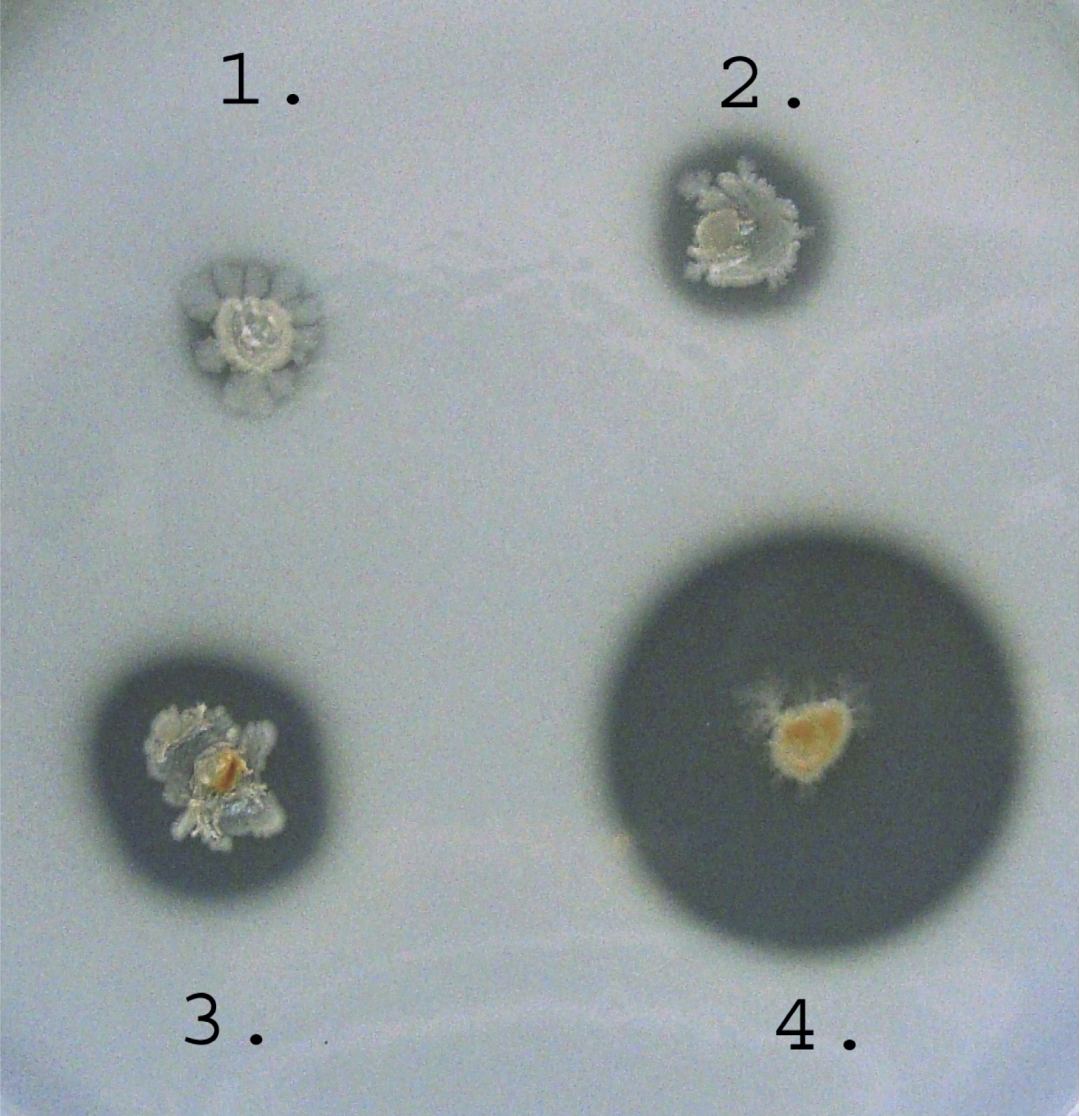
**Cells plated on skim-milk test plates: The dependence of extracellular protease activity on the copy number of the integrated *mpr^B.amy ^*cassettes**. **1**.-clone of *B. subtilis *JE852; **2**. and **3**.-1 and 3 copies of *mpr^B.amy^*-cassette integrated in the chromosome of JE852; **4**.-clone of the plasmid-carrier strain *B. subtilis *JE852/pHE52*mpr*.

**Figure 4:**
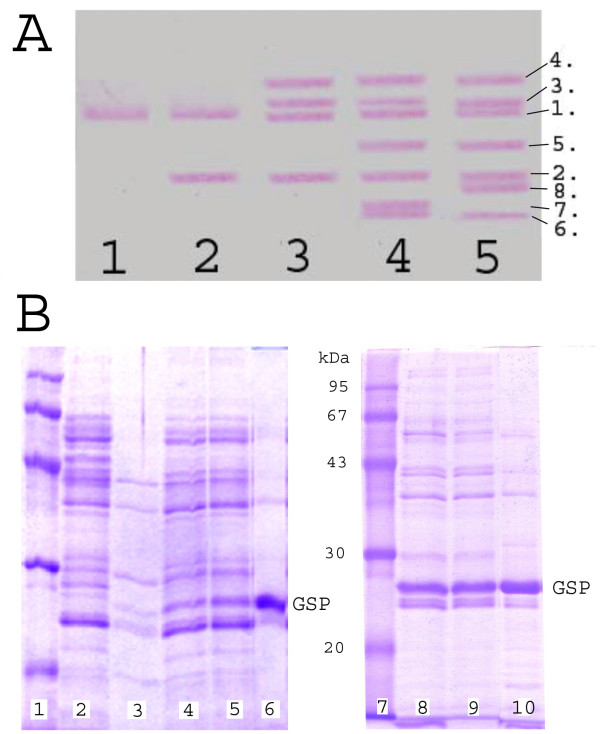
**Southern hybridization of the chromosomal DNAs and SDS-PAGE of extracellular proteins secreted by the strains with different copy-number (N) of the integrated mprB.amy-cassettes.A** – Hybridization with DNAs isolated from cells carrying (indicated as 1.; 2.; 3; etc. (N = 1: lane 1 (1.); N = 2: lane 2 (1.+2.); N = 4: lane 3 (1.+2.+3.+4.); and N = 7: lane 4 (1.+2.+3.+4.+5.+6.+7.) and lane 6 (1.+2.+3.+4.+5.+6.+8.). **B **SDS-PAGE of extracellular proteins secreted by the strains with: N = 0 (lane 2), N = 1 (lane 3), N = 2 (lane 4), N = 3 (lane 5), N = 9 (lane 8) and N = 10 (lane 9) *mpr^B.amy ^*cassettes in the chromosome or carrying the multi-copy-number recombinant plasmid pHE52*mpr *(lanes 6 and 10). Lanes 1 and 7-reference proteins with molecular mass given in kDa. The mature 220 amino acid *B. amiloliquefaciens *GSP has a molecular mass of about 23.7 kDa. The indicated extracellular GSP presented slightly lower electrophoretic mobility corresponding to 27-28 kDa; this was previously documented for the mature GSP from *B. subtilis *[17].

The dependence of GSP accumulation on the integrated cassette copy-number (**N**) was evaluated according to the semi-quantitative plate test based on casein hydrolysis (Figure 3) and using SDS-PAGE analysis of extracellular bacterial proteins (Figure 4). The results showed that GSP production was significantly lower than that of a recombinant strain that had multiple plasmid copies, even for the plasmid-less strain, which had three cassette insertions (**N **= 3). This finding suggested that the process of cassette amplification needed to be continued. However, simplifying the procedure to obtain many single-copy integrants and then combining the variants possessing segregation stability became an attractive option.

### Integration of the *mpr^B.amy ^*cassette into random sites in the bacterial genome

**Figure 5:**
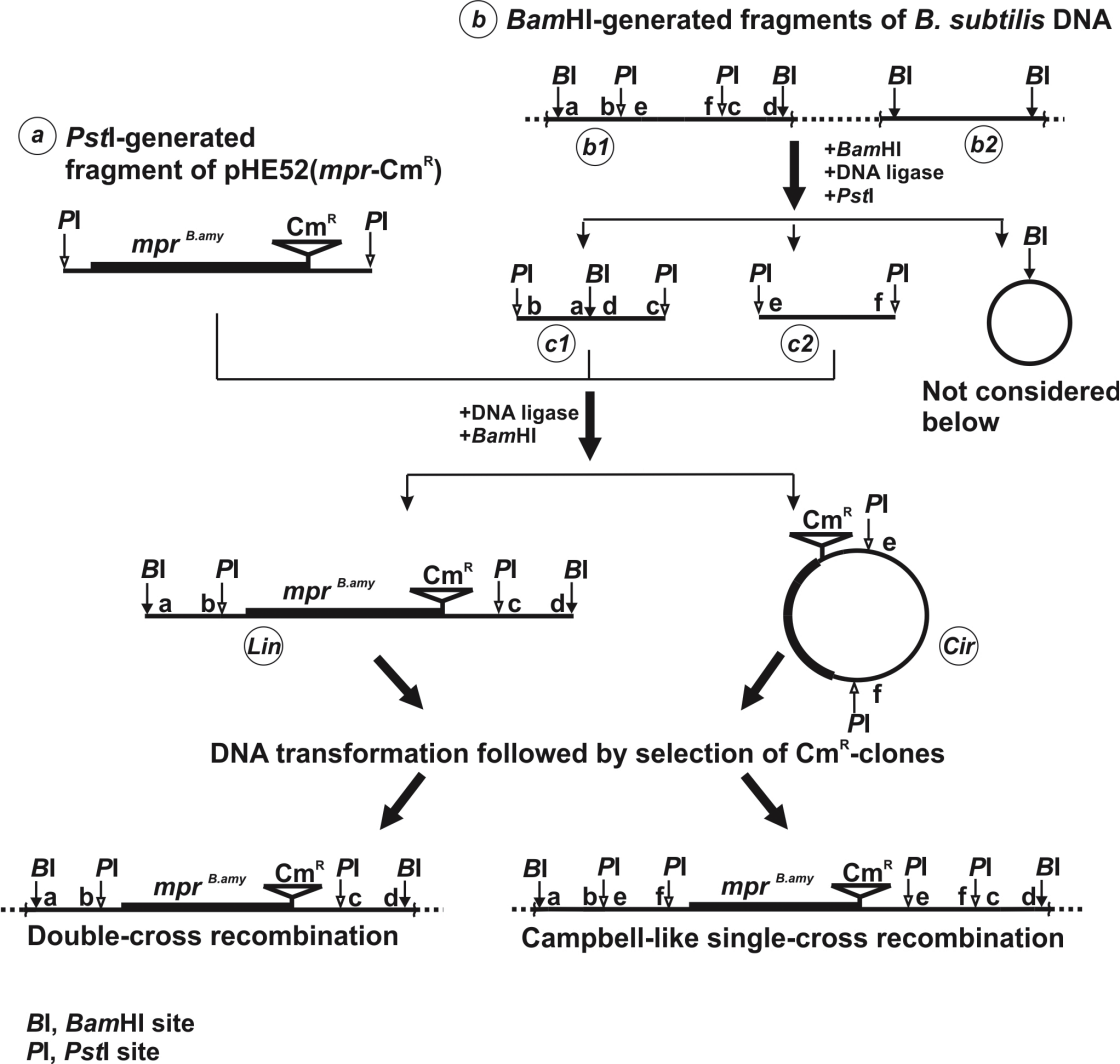
**The general scheme of the *mpr^B.amy^*-Cm^R^-cassette integration into random points of the *B. subtilis *chromosome**.

A key aspect of the novel strategy presented here is the construction of DNA fragments in which the AntR-marked cassette (*mpr^B.amy^*-AntR) is sandwiched between the "front" and "back" portions of randomly digested fragments of the recipient chromosome. The proposed scheme is presented in Figure 5. Initially, pHE52(*mpr*-Cm^R^) was constructed (see **Materials and methods**). This plasmid carried the *mpr^B.amy^*-Cm^R ^cassette that was bracketed by *Pst*I-sites and did not contain internal *Bam*HI-sites. The *Pst*I-generated *mpr^B.amy^*-Cm^R ^cassette is marked as (***a***) in Figure 5. The *Bam*HI-generated DNA fragments of the *B. subtilis *JE852 chromosome ((***b***) fragments in Figure 5) were self-circularized by T4 ligase at a low DNA concentration and subsequently cleaved by *Pst*I. (***b***)-fragments in Figure 5 were a mixture of *Pst*I-site(s)-carrying (***b1***) and *Pst*I-site-free (***b2***) fragments. The (***b1***)-fragment with two internal *Pst*I-sites was shown in the Figure 5 for simplicity. The self-circularized (***b2***) fragments could not be linearized by *Pst*I and so would not be later linked with the (***a***)-fragment. In contrast, the self-circularized (***b1***)-fragments hydrolyzed by *Pst*I generated a mixture of *Bam*HI-site-carrier (***c1***) and *Bam*HI-site-free (***c2***) linear DNA fragments. The ligation of (***c1***)-fragments with (***a***)-fragment followed by *Bam*HI treatment caused formation of linear (***Lin***) fragments consisting of the cassette of interest sandwiched by "front" and "back" homologous arms. These (***Lin***)-fragments could participate in subsequent double-cross recombination-mediated integration into the bacterial chromosome. (***c2***)-fragments could be ligated with the (***a***)-fragment as well. These circular, recombinant DNAs, (***Cir***)-fragments, were resistant to *Bam*HI-mediated cleavage and could be integrated into the chromosome only via a single-cross Campbell-type recombination. It could be supposed that the number of (***Cir***)-mediated integrants would be more than (***Lin***)-mediated ectopic insertions [10]. At the same time, the (***Cir***)-mediated integrant with the cassette sandwiched between directly repeated (***c2***)-fragments (see Figure 5) could be rather unstable due to the possibility of recombination-dependent elimination of the cassette.

The success of the strategy led to the formation of about 250 Cm^R ^clones after transformation of the *B. subtilis *JE852 strain. These colonies were tested for their ability to grow on minimal media with glucose as the sole carbon source. Prototrophic Cm^R ^strains (190 colonies) were then tested for their segregation stability (see **Materials and methods **for details). Fifteen transformants that demonstrated 100% segregation stability after 60 generations were used in the experiments that followed. According to data from the literature [10], it could be supposed that the stable integrants were obtained due to the intrinsic ectopic insertions, whereas transformants that manifested decreased segregation stability were the result of Campbell-type integration.

According to experimental evaluation (including growth on skim milk plates and SDS-PAGE analysis of extracellular proteins), all 15 stable integrants produced and secreted GSP at slightly variable levels, and the levels corresponded to the presence of one *mpr^B.amy ^*cassette in the chromosome of *B. subtilis *JE852. Testing by Southern hybridization confirmed that these strains carried only one *mpr^B.amy^*-Cm^R ^cassette integrated into different chromosomal loci (see Figure 4 where results for the corresponding marker-free *mpr^B.amy^*-cassettes were presented). The strains from this set were designated, for example, *B. subtilis *JE852-(69*xyz*::*mpr^B.amy^*-Cm^R^). Here the number, 69 (84, 85, 114, *etc*. for the other strains), indicates the strain number in the laboratory collection, while the uniform three-letter appellation for all strains, *xyz*, indicates that the location of the cassette integration was not determined.

### Step-by-step increase of the *mpr^B.amy ^*cassette chromosomal copy number

The set of strains with integrated *mpr^B.amy^*-Cm^R ^cassettes was used to increase the occurrence of the *mpr^B.amy ^*gene in the genome of a strain that initially possessed three cassettes, *B. subtilis *JE852(*aprE851, epr, nprB*)::*mpr^B.amy^*. For cassette amplification, extracted chromosomal DNA from one Cm^R ^strain was used for transformation of a marker-less strain carrying **N **copies of the *mpr^B.amy^*-cassette (initially **N **= 3 in these experiments). Selection of Cm^R ^transformants led to the creation of a *B. subtilis *genome that contained **N+1 **copies of the gene encoding GSP. At the last step of this round of cassette amplification, the strain was rendered marker-less by gene conversion with pHE52*mpr *followed by plasmid curing. Then, the next Cm^R^-marked cassette was inserted into the chromosome of the newly obtained strain, which carried **N+1 **cassettes.

The level of GSP secretion and the presence of all previously integrated cassettes in the bacterial genome were assessed at each stage of the cassette amplification process using Southern hybridization and SDS-PAGE analysis of extracellular proteins (Figure 4). As a rule, each subsequent generation displayed a slightly increased level of GSP accumulation in comparison to the previous generation, maintained the earlier integrated cassettes at their original positions in the bacterial genome and presented one novel hybridized DNA fragment that could be detected in the marker-less derivative of the corresponding donor strain.

Ultimately, a plasmid-less and marker-less strain carrying 10 copies of the *mpr^B.amy ^*cassette was obtained. This strain efficiently secreted GSP at the same level as the control, *B. subtilis *JE852/pHE52*mpr*.

## Conclusion

Efficient production and secretion of *B. amyloliquefaciens *A-50 GSP by a recombinant plasmid-less *B. subtilis *strain was obtained. The mutant *B. subtilis *JE852 (*nprE, aprE*), which possessed significantly decreased levels of major extracellular proteases, was utilized as the initial recipient strain. The *mpr^B.amy ^*cassette, in which transcription of the *mpr *gene was controlled through a promoter that drives genes for ribosomal proteins in combination with a Rho-independent terminator, was expressed and stably maintained. Finally, the *mpr^B.amy ^*cassette was amplified by multiple ectopic insertions of the construct into the *B. subtilis *chromosome within known genes initially and then in random loci according to the methodology described above. The methods used for these insertions differed slightly but used the following general steps: (*i*) an AntR-marked linear DNA fragment was sandwiched between two arms that were homologous to a target in the bacterial genome, (*ii*) this fragment was incorporated using double homologous recombination, (*iii*) the marker was removed by gene conversion between the chromosome and an introduced plasmid and (*iv*) the incoming plasmid was eliminated from the cell.

It should be mentioned that the efficiency of iterative gene conversions using the same plasmid, pHE52*mpr*, decreased slightly with an increase in the copy number of the integrated cassettes. This efficiency was about (3 to 4)% when **N **= 3 or 4 but did not exceed 1% for strains with **N **= 9 or 10. Excisable markers that can be efficiently removed by different site-specific recombination events [27,51] might be preferable for the amplification procedure.

The constructed plasmid-less strain, which has 10 chromosomal *mpr^B.amy ^*cassettes, displayed essentially the same GSP production level as the recombinant plasmid-carrying strain. According to data in the literature, there are likely 20-30 copies of the pSM19035 replicon-based plasmid in the recombinant plasmid-carrier strain [10,52,53]. This apparent incongruity has several explanations. First, the copy number of the recombinant *mpr^B.amy^*-carrier plasmid could be lower than that of the vector, in particular, because of interference between plasmid replication and efficient intra-plasmid transcription. Second, expression levels of the same gene located in the chromosome vs. located on a plasmid could differ due to changes in the DNA curvature; dependence on restrained superhelical density is typical of protein-bound DNA molecules [54]. Third, P*_rp_*-mediated transcription of even ten copies of the *mpr *gene may be inherently efficient, such that the saturated translation/secretion machinery becomes the true bottleneck for extracellular GSP accumulation.

Segregation stability is a major factor that must be considered in the potential practical application of plasmid-less recombinant strains. As mentioned previously, only 10% of the clones that had a single-copy of the *mpr^B.amy^*-carrying cassette integrated at random points within the bacterial chromosome possessed strong segregation stability. Amplification of the same cassettes in one genome could certainly decrease the strain's stability due to the potential for homologous intrachromosomal recombination. Recombination between directly repeated cassettes can lead to internal chromosomal deletions such that the strains, possessing essential genes in regions between the cassettes, have to be protected from these genomic rearrangements. In turn, recombination between inversely repeated cassettes leading to chromosomal inversions [55,56] could be the basis of strain instability and, in particular, the decreased performance of the corresponding strain.

It seems useful to determine the integration points to finalize the construction of a set of stably-maintained single-copy cassette integrants. This determination could be performed using inverse PCR-based methods [57]. In this case, a task-oriented amplification of the cassettes could be performed to exclude the formation of inverted repeats and to localize essential genes between directly repeated cassettes.

It is possible that this strategy of ectopic multi-copy integration would be helpful for the construction of a broad range of plasmid-less, marker-less, recombinant *Bacillus *strains for microbial technology applications.

## Materials and methods

### Bacterial strains, plasmids, and culture conditions

Strains and plasmids used in the present study are shown in Additional file 3, **Table S2**. Cells of *B. amyloliquefaciens *and *B. subtilis *were grown at 37°C in liquid LB media or LB with agar [58] supplemented by antibiotics (chloramphenicol (Cm, 5-10 mg/L) or erythromycin (Em, 10 mg/L) when necessary.

Cells were plated on skim milk (20%) test plates for semi-quantitative detection of the total extracellular protease activity; activity was determined by the size of the clearance zone around each colony [17,22].

The fermentation media TYS6C that was used for GSP production was composed of the following: 2% tryptone, 3% yeast extract, 6% soluble starch, 2% corn steep liquor (CSL), 0.1% CaCl_2 _(added after autoclaving), and 1% CaCO_3 _(added after sterilization) at pH 7.0. A final concentration of 10 mg/L Em was added to the media for cultivation of the plasmid-carrying strain. *B. subtilis *strains were cultured for 48 hours on a rotary shaker (at 220 rpm) at 37°C in 750-mL flasks containing 30 mL of media. Seed cultures were standardized by the preparation of freezer stock (-70°C) cultures in 20% glycerol. Then, 0.15 mL of the seed culture from the glycerol stock was used to inoculate 30 mL of TYS6C media in a single 750-mL flask. Samples for SDS-PAGE were taken after 48 hours of bacterial cultivation.

TYS6 media was the same as TYSC media, but without the CSL component. TYS6 media with 2%-4% glucose or maltose was used as the test media for generating CCR conditions.

### Standard genetic engineering methods

Transformation of *B. subtilis *was performed using the method described by Spizizen [59].

Treatment of recombinant DNA and Southern hybridization were carried out in accordance with conventional protocols [60]. Chromosomal DNA of *B. subtilis *strains was hydrolyzed by *Eco*RI overnight, separated by electrophoresis in agarose and hybridized with biotinilated, *mpr*-containing PCR fragments that were amplified with mprF/mprR primers using pHE52*mpr *as a template. The Biotin DecaLabel™ Kit and Biotin Chromogenic Detection Kits (Fermentas, Lithuania) were used to label and detect DNA.

Preparations of restriction enzymes, T4 DNA ligase and DNA polymerase I Klenow fragments from Fermentas were used. Taq DNA polymerase (Fermentas) or AccuTaqLA DNA polymerase (Sigma, USA) were used for PCR in accordance with the manufacturers' instructions. The structures of all primers used in the present study are listed in Additional file 1, **Table S1**.

### Construction of the pHE52*mpr *and pHE52(*mpr*-Cm^R^) plasmids

The pairs of primers mprF/mprR and P1-bmp5/P2-bmp2 were used for PCR-mediated amplification and then for cloning of the *mpr *gene from the chromosomal DNA of *B. amyloliquefaciens *A-50. The amplicons, generated in PCR with P1-bmp5/P2-bmp2 as the primers, were treated with *Bgl*II and inserted into the *Bgl*II site of the pHEA323 plasmid [40] to form the pHE52*mpr *plasmid. The Cm^R ^gene from the pC194 plasmid [50] was cloned into a *Bgl*II-site of the pHE52*mpr *plasmid located just downstream of the *mpr *gene (with coordinate (2,174) in Figure 1). As a result, the pHE52(*mpr*-Cm^R^) plasmid carrying the *mpr^B.amy^*-Cm^R ^cassette was obtained. The *mpr^B.amy ^*and *mpr^B.amy^*-Cm^R ^cassettes had the mutual DNA fragments not only in proximal part, but in distal part, as well. The later included *B. amyloliquefaciens *DNA fragment of the pHE52*mpr *plasmid (about 1,800 bp in length) consisted of *pheA *gene and Ter. So, pHE52*mpr *plasmid could be efficiently used for gene conversion resulting in substitution of *mpr^B.amy^*-Cm^R ^cassette integrated in the chromosome by the marker-less *mpr^B.amy^*-cassette from the plasmid (see below).

### Construction of the JE852(*aprE851, epr, nprB*)::*mpr^B.amy ^*strain

The strain JE852(*aprE851, epr, nprB*)::*mpr^B.amy ^*was constructed via step-by-step ectopic integration of three copies of the *mpr^B.amy ^*cassette into the *aprE851, epr *and *nprB *genes of the JE852 strain. For each integration, two target-specific DNA molecules were constructed: (*i*) linear Cm^R^-carrier DNA fragments for the target gene inactivation and (*ii*) *mpr^B.amy^*-carrier plasmids for gene conversion.

As for integration into the *aprE851 *gene, the linear DNA fragment, *yhfO'*-Cm^R^-'*yhfN*, was constructed *in vitro *by overlapping PCR, as shown in Additional file 2, **Figure S1**. The final DNA amplicon was treated with *Eco*RI and cloned into a pCB20-based [52] plasmid for the construction of pCBT(*yhfO*-Cm^R^-*yhfN*). The later recombinant plasmid was used as a vector for the *in vitro *substitution of the Cm^R^-marker by the *Pst*I-generated *mpr^B.amy^*-cassette from pHE52*mpr *(Figure 1). The obtained pCBT(*yhfO*-*mpr^B.amy^*-*yhfN*) plasmid was used for *in vivo *gene conversion, which resulted in construction of the JE852*aprE851*::*mpr^B.amy ^*strain (Figure 2).

A linear DNA fragment for integration into the *epr *gene was designed using Pr7/Pr8 as the primers for PCR-mediated amplification of the *B. subtilis *168 chromosome. Insertion of the *Pst*I-generated amplicon with the Cm^R ^gene from pC194 (the primers-Pr9/Pr10) was between two *Pst*I-sites in the *epr *gene. Two auxiliary plasmids, pCBT-*epr *and pCBT(*epr*::Cm^R^), were obtained for construction of this linear fragment. The latter plasmid served as a vector for the cloning of the *mpr^B.amy ^*cassette from pHE52*mpr*, resulting in pCBT(*epr-mpr*52). The linear *epr*::Cm^R ^DNA fragment and pCBT(*epr-mpr*52) were used for integration of the second copy of the *mpr^B.amy ^*cassette and construction of the JE852(*aprE851, epr*)::*mpr^B.amy ^*strain.

The third integration was based on the linear DNA fragment, *nprB*::Cm^R^, carrying the *nprB *gene (the primers-Pr11/Pr12) disrupted by a *Hind*III-generated Cm^R^-carrier amplicon from pC194 (primers-Pr9/Pr10) that was inserted into the unique *Hind*III site in the structural part of *nprB*. Construction of this fragment was provided through formation of the auxiliary plasmid pCBT(*nprB*::Cm^R^). This plasmid was used later as a vector for cloning of the *Pst*I-generated *mpr^B.amy ^*cassette instead of Cm^R ^disrupted of *nprB *and construction of pCBT(*nprB*-*mpr*52). It was possible so long as the Pr9/Pr10 were designed for bracketing the Cm^R^-marker by (*Hind*III-*PstI*)/(*Pst*I-*Hind*III) sites. The linear DNA fragment, *nprB*::Cm^R^, and the pCBT(*nprB*-*mpr*52) plasmid were used for construction of the JE852(*aprE851, epr, nprB*)::*mpr^B.amy ^*strain that possessed three copies of the *mpr^B.amy^*-cassette in the targeted loci of the bacterial chromosome.

### Construction of DNA fragments for random integration of the *mpr^B.amy^*-cassette

A total of 5 μg of chromosomal DNA from *B. subtilis *JE852 was exhaustively hydrolyzed by *Bam*HI, followed by self-circularization of the linear DNA fragments by treatment with T4 ligase in 1 mL of reaction mixture. This DNA was then digested by *Pst*I and ligated with 5 μg of *Pst*I-generated *mpr^B.amy^*-Cm^R^-cassette from pHE52(*mpr*-Cm^R^) that had been purified from low melting agarose. The ligation mixture was digested by *Bam*HI, and about 1 μg of the total DNA was used for the transformation of *B. subtilis *JE852.

### Segregation stability test

About 10^2 ^cells from overnight cultures of the *B. subtilis *JE852-(N*xyz*::*mpr^B.amy^*-Cm^R^) strains were inoculated into 10 mL of fresh LB medium, cultivated for 20 generations and cloned. One hundred individual colonies were tested for Cm resistance. Strains that generated 100% Cm^R ^clones after 20 generations were tested for stability after 40 generations and then again after 60 generations. Finally, JE852-(N*xyz*::*mpr^B.amy^*-Cm^R^) strains, which generated 100 Cm^R ^colonies among the 100 that were tested after 60 generations, were considered to be stable and were used as donors of chromosomal DNA for increasing the *mpr^B.amy^*-cassette copy-number.

### Protein analysis

SDS-PAGE was conducted using Laemmli's method [61] for the evaluation of GSP accumulation in the culture supernatants of *B. subtilis *strains. Gels were stained with Coomassie R-250 and scanned to estimate the protein content with the TotalLab v. 2.01 computer software for determine the portion of GSP among the secreted proteins. Total extracellular protein concentrations were determined using the Bio-Rad Protein Assay (Bio-Rad, USA) in accordance with the manufacturer's instructions. In addition, the known concentrations of the commercially available carbonic anhydrase from bovine erythrocytes (Sigma) with Mw 29 kDa were used for SDS-PAGE followed by staining and scanning the gel for comparative evaluation of GSP production. Both independent methods gave, practically, coincident results.

## Abbreviations

AntR: antibiotic resistance marker; AprE: alkaline serine protease subtilisin; bp: base pair(s); CCA: carbon catabolite activation; CCC: carbon catabolite control; CCR: carbon catabolite repression; Cm: chloramphenicol; Cm^R^: Cm resistance; *cre*: catabolite responsive element; CSL: corn steep liquor; Em: erythromycin; Em^R^: Em resistance; GSP: glutamyl-specific protease, the *mpr *gene protein product; marker-less strain: a bacterial strain that does not carry AntR in its genome; NprE: neutral protease; PCR: polymerase chain reaction; P*_rp_*: promoter of the *B. amyloliquefaciens *A-50 *rplU-rpmA *genes; Ter: transcription terminator of the *B. amyloliquefaciens pheA *gene; *mpr^B.amy ^*cassette: expression cassette where the structural portion of the *B. amyloliquefaciens *A-50 *mpr *gene is sandwiched between P*_rp _*and Ter; SDS-PAGE: sodium dodecyl sulphate polyacrilamide gel electrophoresis;/: denotes a plasmid-carrying strain.

## Competing interests

The authors declare that they have no competing interests.

## Authors' contributions

YAVY designed the methods and performed the multi-copy number integrations at random sites of the *B. subtilis *chromosome. EAG designed and constructed the recombinant DNA used in this study and drafted the manuscript. LIG tested the level of extracellular GSP accumulation by protein electrophoresis and edited the manuscript. LYG performed the Southern hybridization experiments. SVM coordinated the work and amended the manuscript. All authors read and approved the final version of the manuscript.

## Supplementary Material

Additional file 1 **Table S1**. List of primers used for PCR. Click here for file

Additional file 2 **Figure S1**. Construction of the linear DNA fragment used for the JE852*aprE::mpr*^*B.amy*^strain construction. Click here for file

Additional file 3 **Table S2**. Bacterial strains and plasmids used. Click here for file
