# Supplementary material for: Overproduction of Bacillus amyloliquefaciens extracellular glutamyl-endopeptidase as a result of ectopic multi-copy insertion of an efficiently-expressed mpr gene into the Bacillus subtilis chromosome
Source: Microb Cell Fact. 2011 Aug 5;10:64. doi: 10.1186/1475-2859-10-64 (PMC3166918; doi:10.1186/1475-2859-10-64)
Supplement: Additional file 1 — Table S1. List of primers used for PCR. [file 1475-2859-10-64-S1.PDF]

Table S1 List of the primers used for PCR

Appellation	Sequence of the primers	Restriction site(s)	For cloning
mpr-F<br>mpr-R	5’-ttaaaacgaaagccggtagacaa-3’<br>5’-tattgatttgaccaatattgaatg-3’	none	a *mpr*-containing DNA fragment from *B. amiloliquefaciens* A-50
P1-bmp5<br>P2-bmp2	5’-atatgtagatctttaaaccgaaagccggtagacaa-3’<br>5’-tcgtagaattcagatcttattgatttgaccaatattgaatg-3’	*Bgl*II<br>*Bgl*II	*B. amiloliquefaciens* A-50 *mpr* gene
Pr.1<br>Pr.2	5’-gcctatgaattctccattttcttctgctatc-3’<br>5’-ttctcactcgagaccctctccttttaaaaaaattcag-3’	*Eco*RI<br>*Xba*I	*yhfO’*-carrier DNA fragment from *B. subtilis* chromosome
Pr.3<br>Pr.4	5’-ggagattctagactgcagccggcaatagttacccttat-3’<br>5’-agttatcggatcctgcagggatctggagctgtaatata-3’	*Xba*I–*Pst*I<br>*Bam*HI– *Pst*I	Cm[R] gene from pC194 plasmid
Pr.5<br>Pr.6	5’-gcacaaggatcctaaaaagaagcaggttcctccatac-3’<br>5’-cagtatgaattcatcggggtaccacttgattggtttc-3’	*Bam*HI<br>*Eco*RI	*‘yhfN*-carrier DNA fragment from *B. subtilis* chromosome
Pr.7<br>Pr.8	5’-ggcattgaattctcctccatcacccgagtgaatgtg-3’<br>5’-ggcattgaattctcctccatcacccgagtgaatgtg-3’	*Eco*RI<br>*Eco*RI	*B. subtilis epr* gene
Pr.9<br>Pr.10	5’-cctcgaagcttctgcagccggcaatagttacccttat-3’<br>5’-gatataagcttctgcagggatctggagctgtaatata-3’	*Hind*III–*Pst*I<br>*Hind*III–*Pst*I	Cm[R] gene from pC194 plasmid
Pr.11<br>Pr.12	5'-gcatgacatatgtattactggccggcttatgcacagc-3'<br>5'-cagtcaggtacctgaaacacctgattgaggaagcgtt-3'	*Nde*I<br>*Kpn*I	*B. subtilis nprB* gene
