# Supplementary material for: Overproduction of Bacillus amyloliquefaciens extracellular glutamyl-endopeptidase as a result of ectopic multi-copy insertion of an efficiently-expressed mpr gene into the Bacillus subtilis chromosome
Source: Microb Cell Fact. 2011 Aug 5;10:64. doi: 10.1186/1475-2859-10-64 (PMC3166918; doi:10.1186/1475-2859-10-64)

Figure S1 Construction of the linear DNA fragment used for the JE852*aprE*::*mpr*$^{B.amy}$ strain construction

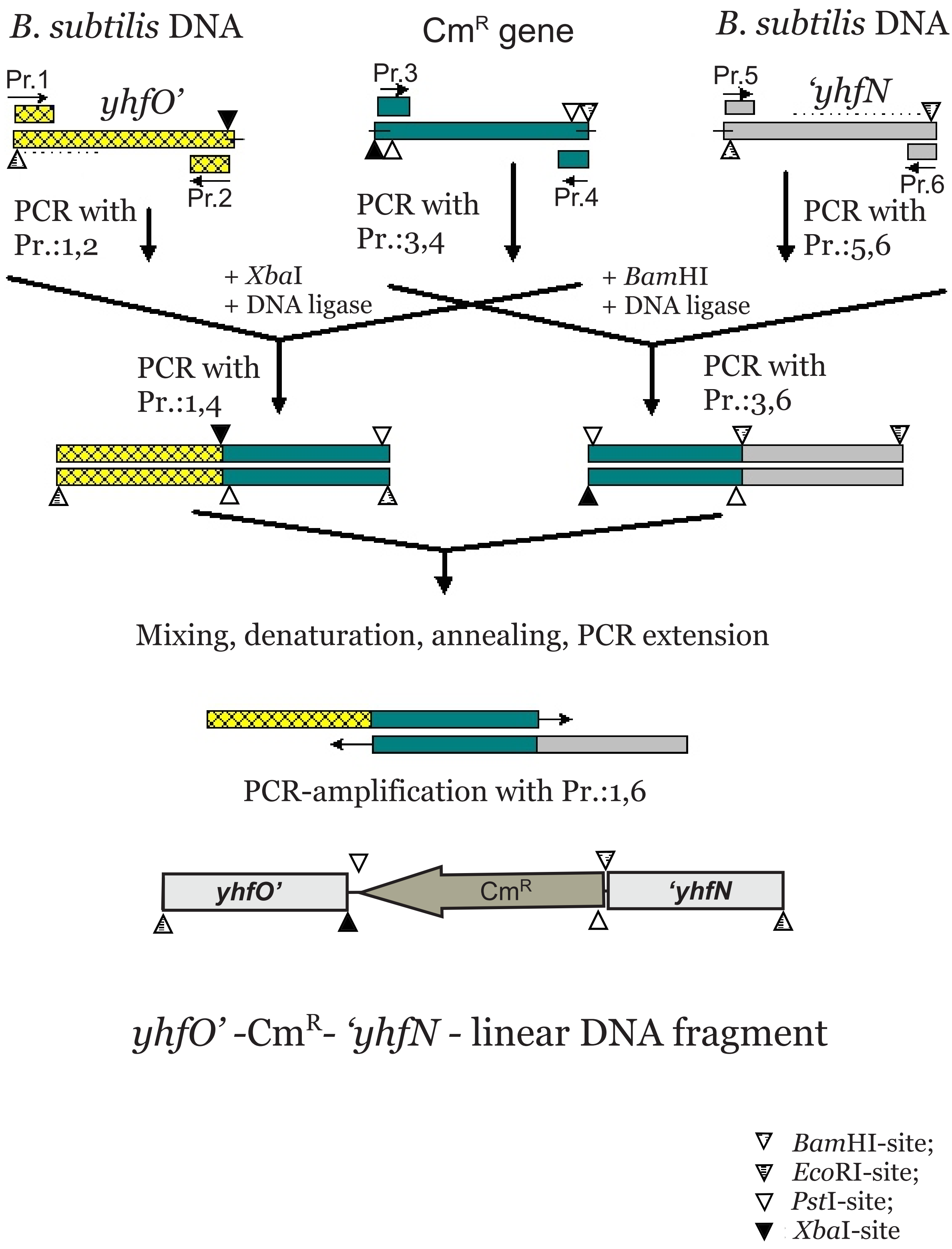

Supplement: Additional file 2 — Figure S1. Construction of the linear DNA fragment used for the JE852aprE::mprB.amystrain construction. [file 1475-2859-10-64-S2.PDF]
