# Supplementary material for: Overproduction of Bacillus amyloliquefaciens extracellular glutamyl-endopeptidase as a result of ectopic multi-copy insertion of an efficiently-expressed mpr gene into the Bacillus subtilis chromosome
Source: Microb Cell Fact. 2011 Aug 5;10:64. doi: 10.1186/1475-2859-10-64 (PMC3166918; doi:10.1186/1475-2859-10-64)
Supplement: Additional file 3 — Table S2. Bacterial strains and plasmids used. [file 1475-2859-10-64-S3.PDF]

Table S2 Bacterial strains and plasmids used

Strains and plasmids	***Relevant characteristics***	***Reference or source***
Strains		
B. amyloliquefaciens A-50		VKPM B5253
B. subtilis strains		
JE852	*nprE512, aprE851*	Laboratory collection
JE852*aprE*::Cm^R	*nprE512, aprE*::Cm^R	This work
JE852*aprE851*::*mpr*$^{B.amy}$	*nprE512, aprE*::*mpr*$^{B.amy}$ (i.e., ::(P$_{rp}$-*mpr*-ter$_{pheA}$)	This work
JE852*aprE851*::*mpr*$^{B.amy}$ *epr*::Cm^R	*nprE512, aprE*::*mpr*$^{B.amy}$, *epr*::Cm^R	This work
JE852(*aprE851, epr*)::*mpr*$^{B.amy}$	*nprE512*, (*aprE, epr*)::*mpr*$^{B.amy}$	This work
JE852(*aprE851, epr*)::*mpr*$^{B.amy}$ *nprB*::Cm^R	*nprE512*, (*aprE, epr*)::*mpr*$^{B.amy}$ *nprB*::Cm^R	This work
JE852-3*mpr*$^{B.amy}$ = JE852(*aprE851, epr, nprB*)::*mpr*$^{B.amy}$	*nprE512*, (*aprE, epr, nprB*)::*mpr*$^{B.amy}$	This work
JE852-(69*xyz*:: *mpr*$^{B.amy}$-Cm^R)	*nprE512, aprE851*, 69*xyz*::*mpr*$^{B.amy}$-Cm^R (i.e., ::(P$_{rp}$-*mpr*-Cm^R-ter$_{pheA}$))	This work
JE852-(84*xyz*:: *mpr*$^{B.amy}$-Cm^R)	*nprE512, aprE851*, 84*xyz*::*mpr*$^{B.amy}$-Cm^R	This work
JE852-(85*xyz*:: *mpr*$^{B.amy}$-Cm^R)	*nprE512, aprE851*, 85*xyz*::*mpr*$^{B.amy}$-Cm^R	This work
JE852-(114*xyz*:: *mpr*$^{B.amy}$-Cm^R)	*nprE512, aprE851*, 114*xyz*::*mpr*$^{B.amy}$-Cm^R	This work
JE852-(133*xyz*:: *mpr*$^{B.amy}$-Cm^R)	*nprE512, aprE851*, 133*xyz*::*mpr*$^{B.amy}$-Cm^R	This work
JE852-(142*xyz*:: *mpr*$^{B.amy}$-Cm^R)	*nprE512, aprE851*, 142*xyz*::*mpr*$^{B.amy}$-Cm^R	This work
JE852-(159*xyz*:: *mpr*$^{B.amy}$-Cm^R)	*nprE512, aprE851*, 159*xyz*::*mpr*$^{B.amy}$-Cm^R	This work
JE852-4*mpr*$^{B.amy}$ = JE852-3*mpr*$^{B.amy}$-(69*xyz*:: *mpr*$^{B.amy}$)	*nprE512*, (*aprE, epr, nprB*, 69*xyz*)::*mpr*$^{B.amy}$	This work
JE852-5*mpr*$^{B.amy}$ = JE852-4*mpr*$^{B.amy}$-(84*xyz*:: *mpr*$^{B.amy}$)	*nprE512*, (*aprE, epr, nprB*, 69*xyz*, 84*xyz*)::*mpr*$^{B.amy}$	This work
JE852-6*mpr*$^{B.amy}$ = JE852-5*mpr*$^{B.amy}$-(85*xyz*:: *mpr*$^{B.amy}$)	*nprE512*, (*aprE, epr, nprB*, 69*xyz*, 84*xyz*, 85*xyz*)::*mpr*$^{B.amy}$	This work
JE852-6*mpr*$^{B.amy}$ = JE852-5*mpr*$^{B.amy}$-(85*xyz*:: *mpr*$^{B.amy}$)	*nprE512*, (*aprE, epr, nprB*, 69*xyz*, 84*xyz*, 85*xyz*)::*mpr*$^{B.amy}$	This work
JE852-7*mpr*$^{B.amy}$ = JE852-6*mpr*$^{B.amy}$-(114*xyz*:: *mpr*$^{B.amy}$)	*nprE512*, (*aprE, epr, nprB*, 69*xyz*, 84*xyz*, 85*xyz*, 114*xyz*)::*mpr*$^{B.amy}$	This work
JE852-8*mpr*$^{B.amy}$ = JE852-7*mpr*$^{B.amy}$-(133*xyz*:: *mpr*$^{B.amy}$)	*nprE512*, (*aprE, epr, nprB*, 69*xyz*, 84*xyz*, 85*xyz*, 114*xyz*, 133*xyz*)::*mpr*$^{B.amy}$	This work
JE852-9*mpr*$^{B.amy}$ = JE852-8*mpr*$^{B.amy}$-(142*xyz*:: *mpr*$^{B.amy}$)	*nprE512*, (*aprE, epr, nprB*, 69*xyz*, 84*xyz*, 85*xyz*, 114*xyz*, 133*xyz*, 142*xyz*)::*mpr*$^{B.amy}$	This work
JE852-10*mpr*$^{B.amy}$ = JE852-9*mpr*$^{B.amy}$-(159*xyz*:: *mpr*$^{B.amy}$)	*nprE512*, (*aprE, epr, nprB*, 69*xyz*, 84*xyz*, 85*xyz*, 114*xyz*, 133*xyz*, 142*xyz*, 159*xyz*)::*mpr*$^{B.amy}$	This work
Plasmids		
pCB20	del-pSM19035-pUC18, Em^R, Ap^R	[52]
pCBT20	pCB20 without *Pst*I site, Em^R	This work
pC194	Cm^R, (GenBank/EMBL NC_002013)	[50]
pHEA323	pCB20-(P$_{rp}$-pheA*), Em^R	[40]

pHE52*mpr*	pHEA323-*mpr*$^{B.amy}$, Em^R	This work
pCBT(*yhfO*-[*Pst*I]-*yhfN*)	pCBT20-(*yhfO*-[*Pst*I]-*yhfN*), Em^R	This work
pCBT(*yhfO*-Cm^R-*yhfN*)	pCBT20-(*yhfO*-Cm^R-*yhfN*), Em^R	This work
pCBT(*yhfO-mpr*$^{B.amy}$-*yhfN*)	pCBT20-(*yhfO-mpr*$^{B.amy}$-*yhfN*), Em^R	This work
pHE52(*mpr*-Cm^R)	pHEA323-(*mpr*$^{B.amy}$-Cm^R), Em^R	This work
pCBT(*epr*::Cm^R)	pCBT20-*epr*::Cm^R, Em^R	This work
pCBT(*epr-mpr*52)	pCBT20-*epr*::*mpr*$^{B.amy}$, Em^R	This work
pCBT(*nprB*::Cm^R)	pCBT20-*nprB*::Cm^R, Em^R	This work
pCBT(*nprB-mpr*52)	pCBT20-*nprB*::*mpr*$^{B.amy}$, Em^R	This work
